# Higher job exposures are associated with reduced return-to-work two years after rehabilitation in a nationwide cohort study based on German Pension Insurance data

**DOI:** 10.1038/s41598-026-55323-0

**Published:** 2026-06-02

**Authors:** Martin Brünger, Paul Gellert

**Affiliations:** 1 Institute of Medical Sociology and Rehabilitation Science, Charité – Universitätsmedizin Berlin, corporate member of Freie Universität Berlin and Humboldt-Universität zu Berlin, Berlin, Germany; 2 Einstein Center Population Diversity (ECPD), Berlin, Germany

**Keywords:** Return-to-work, Job exposure, Job demands, Rehabilitation, Occupational health, Routine data, Health care, Medical research, Risk factors

## Abstract

Return-to-work (RTW) after medical rehabilitation is an important indicator of post-rehabilitation labour market participation, yet evidence on its association with occupation-based job exposure over longer follow-up periods remains limited. This nationwide retrospective cohort study examined the association between job exposure and RTW within 24 months post-rehabilitation using routine administrative data from the German Pension Insurance. The study included 621,695 individuals aged 18–63 years who completed medical rehabilitation between 2014 and 2019. Job exposure was assessed using the Overall Job Exposure Index, integrating physical and psychosocial exposures, and categorised into low, moderate, and high exposure. Initial RTW was defined as at least one month of employment, and stable RTW as at least four consecutive months within 24 months post-rehabilitation. Associations were analysed using Cox proportional hazards regression models with progressive adjustment for sociodemographic, work-related, and health-related factors. Within 24 months, 90.3% achieved initial RTW and 84.2% stable RTW. Higher job exposure was consistently associated with lower RTW rates. In fully adjusted models, moderate and high exposure were associated with a reduced likelihood of initial RTW (HR 0.903 [95% CI 0.897–0.909] and 0.872 [95% CI 0.866–0.878]) and stable RTW (HR 0.878 [95% CI 0.872–0.884] and 0.837 [95% CI 0.830–0.843]), compared with low exposure. These findings provide population-level evidence that occupation-based job exposure is associated with RTW after medical rehabilitation.

**Supplementary Information:**

The online version contains supplementary material available at 10.1038/s41598-026-55323-0.

## Introduction

 Job exposures are closely linked to a wide range of adverse health outcomes and labour market consequences, including mortality^1^, morbidity^2–6^, injury^7,8^, sickness absence^9^, and premature exit from the labour market^10–13^. Previous research has consistently shown that physically demanding working conditions as well as psychosocial stressors such as time pressure, low job control, shift work, or irregular working hours contribute to health-related inequalities and negatively affect participation in working life^14–16^.

Return-to-work (RTW) is a central outcome and a key indicator of rehabilitation success for persons of working age undergoing medical rehabilitation^17,18^. In Germany, medical rehabilitation for persons of working age is primarily provided by the German Pension Insurance, which aims to maintain or restore work ability and to prevent long-term work disability. Rehabilitation measures are typically granted to insured individuals with health impairments that threaten their participation in working life and include multidisciplinary interventions addressing functional capacity as well as work-related factors. As such, the rehabilitation system is closely linked to labour market participation and return-to-work outcomes^19^.

In this context, adverse working conditions are highly relevant, as they may hinder sustainable reintegration into employment after rehabilitation and thereby compromise long-term work participation^17,20^. Despite their importance, information on individual job exposures is rarely available in clinical studies and is not routinely documented in administrative data sources, such as those of the German Pension Insurance^21^. As a result, job-related working conditions are often not considered in large-scale routine data analyses of rehabilitation outcomes^22^.

One approach to address this limitation is the use of job exposure matrices (JEM), which assign typical exposure profiles to occupations based on empirical data collected among employed persons^23^. Many job exposures are strongly associated with specific occupations, allowing occupation to be used as a proxy for job exposures^9,24^. The Overall Job Exposure Index (OJI) was derived from a nationwide survey of 20,000 employed persons in Germany assessing physical and psychosocial job exposures^25^. It combines multiple dimensions of job exposures into an occupation-based index and has been shown to be applicable across different occupational classification systems^26^. This approach enables the assessment of job exposures in routine data analyses whenever information on occupation is available^22^. In two independent studies linking survey data with routine data from the German Pension Insurance, self-reported job exposures among rehabilitation patients corresponded closely with exposure estimates derived from the OJI based on job exposure matrices^27,28^.

A previous study using the OJI has also demonstrated that higher occupation-based job exposure is associated with unfavourable health indicators and with a poorer RTW prognosis at the end of medical rehabilitation^22^. However, RTW prognosis at discharge does not necessarily reflect actual work participation after rehabilitation, and evidence on the association between occupation-based job exposure and realised RTW over longer follow-up periods remains limited, particularly with regard to sustained employment and beyond studies focusing on specific disease groups such as injuries or cancer^20,29^.

Beyond its relevance at the individual level, successful RTW after medical rehabilitation has important implications for social security and the pension system^30,31^. International evidence underscores the relevance of work disability as a persistent labour market outcome: OECD analyses indicate that in many member countries, approximately 1–2% of the working-age population enter disability benefit schemes each year, with only limited chances of subsequent re-entry into employment^31^. In Germany, this corresponds to around 170,000 new disability pensions granted annually^32^. These figures highlight that work disability and sustainable return-to-work are not only individual clinical outcomes but also issues of broader public health and socioeconomic relevance. In this context, high job exposures are of particular concern, as they may limit post-rehabilitation work capacity, reduce the likelihood of sustained employment, and thereby contribute to unfavourable individual and societal outcomes^32^.

Against this background, the present study examines the association between job exposures, operationalised by the Overall Job Exposure Index, and realised return-to-work after medical rehabilitation using routine data of the German Pension Insurance. Specifically, we investigate both initial RTW, defined as the first re-entry into employment, and stable RTW, defined as at least four consecutive months of sustained employment, using monthly employment records over a follow-up period of up to 24 months after rehabilitation discharge.

## Results

The final analytical sample comprised 621,695 insured individuals who completed a first medical rehabilitation between 2014 and 2019 and fulfilled all inclusion criteria. Baseline sociodemographic, work and health-related characteristics of the study population are shown in Table 1. The mean age at rehabilitation was 50.7 years, and 50.3% were female. Most individuals were continuously employed in the year prior to rehabilitation. According to the Overall Job Exposure Index, 25.4% were classified as having low, 38.0% moderate, and 36.5% high job exposure.

**Table 1:** Baseline characteristics stratified by job exposure level.

*n* = 621,695	Total	Low OJI (1–3)	Moderate OJI (4–7)	High OJI (8–10)
	*n* (%) / M ± SD	*n* (%) / M ± SD	*n* (%) / M ± SD	*n* (%) / M ± SD
***Sociodemographic characteristics***				
**Age** (mean, standard deviation, in years)	50.7 (8.9)	50.8 (8.7)	50.7 (9.1)	50.7 (9.0)
**Age groups**				
18–44 years	127,360 (20.5%)	31,892 (20.2%)	49,009 (20.7%)	46,459 (20.5%)
45–49 years	94,945 (14.3%)	24,482 (15.5%)	35,659 (15.1%)	34,804 (15.3%)
50–54 years	147,114 (23.7%)	38,158 (24.1%)	55,000 (23.3%)	53,956 (23.8%)
55–59 years	160,388 (25.8%)	40,431 (25.6%)	60,971 (25.8%)	58,986 (26.0%)
60–63 years	91,888 (14.8%)	23,099 (14.6%)	35,843 (15.2%)	32,946 (14.5%)
**Sex**				
Male	312,835 (50.3%)	61,549 (38.9%)	105,424 (44.6%)	145,862 (64.2%)
Female	308,860 (49.7%)	96,513 (61.1%)	131,058 (55.4%)	81,289 (35.8%)
**Citizenship**				
German	588,471 (94.7%)	154,211 (97.6%)	224,758 (95.0%)	209,502 (92.2%)
Not German	33,224 (5.3%)	3,851 (2.4%)	11,724 (5.0%)	17,649 (7.8%)
**Marital status**				
Single	107,207 (17.2%)	27,883 (17.6%)	40,361 (17.1%)	38,963 (17.2%)
Married	411,084 (66.1%)	103,132 (65.2%)	154,498 (65.3%)	153,454 (67.2%)
Divorced	80,094 (12.9%)	20,349 (12.9%)	32,238 (13.6%)	27,507 (12.1%)
Widowed	15,387 (2.5%)	3,429 (2.2%)	6,273 (2.7%)	5,685 (2.5%)
Unknown	7,923 (1.3%)	3,269 (2.1%)	3,112 (1.3%)	1,542 (0.7%)
**Settlement Structure Type**				
Big urban municipalities	135,233 (21.8%)	40,116 (25.4%)	50,831 (21.5%)	44,286 (19.5%)
Urban districts	248,297 (39.9%)	64,645 (40.9%)	94,806 (40.1%)	88,846 (39.1%)
Rural areas with population concentrations	121,779 (19.6%)	27,632 (17.5%)	46,426 (19.6%)	47,721(21.0%)
Rural areas with a low population density	109,446 (17.6%)	22,835 (14.4%)	41,794 (17.7%)	44,817 (19.7%)
Not living in Germany, or unknown residence	6,940 (1.1%)	2,834 (1.8%)	2,625 (1,1%)	1,481 (0.7%)
***Work- and health-related characteristics***				
**Incapacity for work at rehabilitation start (physician-assessed)**				
No	389,547 (62.7%)	10,6928 (67.6%)	148,256 (62.7%)	134,363 (59.2%)
Yes	232,148 (37.3%)	5,1134 (32.4%)	88,226 (37.3%)	92,788 (40.8%)
**Sick leave duration 12 months before rehabilitation**				
None	71,977 (11.6%)	21,310 (13.5%)	26,922 (11.4%)	23,745 (10.5%)
< 3months	345,480 (55.6%)	92,500 (58.5%)	129,622 (54.8%)	123,358 (54.3%)
3 to < 6 months	104,819 (16.9%)	22,022 (13.9%)	40,290 (17.0%)	42,507 (18.7%)
6 to 12 months	95,590 (15.4%)	21,311 (13.5%)	38,086 (16.1%)	36,193 (15.4%)
Not employed	3,829 (0.6%)	919 (0.6%)	1,562 (0.7%)	1,348 (0.6%)
**Disease group**				
Orthopaedics, rheumatology (including musculoskeletal disorders)	309,640 (49.8%)	69,487 (44.0%)	116,756 (49.4%)	123,397 (54.3%)
Cardiology, angiology	55,367 (8.9%)	13,233 (8.4%)	20,110 (8.5%)	22,024 (9.7%)
Pulmonology	18,443 (3.0%)	4,894 (3.1%)	7,277 (3.1%)	6,272 (2.8%)
Gastroenterology, nephrology, endocrinology	20,133 (3.2%)	5,165 (3.3%)	7,735 (3.3%)	7,233 (3.2%)
Haematology, oncology	45,141 (7.3%)	14,638 (9.3%)	16,865 (7.1%)	13,638 (6.0%)
Neurology	33,338 (5.4%)	9,560 (6.0%)	12,416 (5.3%)	11,362 (5.0%)
Mental diseases	108,383 (17.4%)	33,822 (21.4%)	43,993 (18.6%)	30,568 (13.5%)
Addiction disorder	13,365 (2.1%)	2,195 (1.4%)	4,610 (1.9%)	6,560 (2.9%)
Other diseases	17,885 (2.9%)	5,068 (3.2%)	6,720 (2.8%)	6.097 (2.7%)
***Job exposures***				
**Overall Job Exposure Index (OJI)**				
Low (OJI 1–3)	158,062 (25.4%)			
Moderate (OJI 4–7)	236,482 (38.0%)			
High (OJI 8–10)	227,151 (36.5%)			

OJI: Overall Job Exposure Index; M: mean, SD: standard deviation.

### Initial and stable RTW

Initial RTW, defined as at least one month of employment subject to compulsory social insurance contributions, was achieved by 90.3% of participants, whereas stable RTW, defined as at least four consecutive months of such employment at any time within the 24-month follow-up period, was achieved by 84.2% (Table 2). At 12 months after rehabilitation discharge, 71.9% of participants were in stable employment; after 24 months, this proportion was 69.1% (Table S1).

For all RTW outcomes, RTW rates differed by job exposure level, with lower proportions observed among individuals with higher exposure. Among those with low job exposure, 93.2% achieved initial RTW within 24 months, compared with 89.8% among those with moderate exposure and 88.7% among those with high exposure. A similar gradient was observed for stable RTW, with corresponding proportions of 88.9%, 83.4%, and 81.6% for low, moderate, and high job exposure, respectively (Table 2). Comparable gradients were observed for stable RTW at both 12 and 24 months after rehabilitation discharge (Table S1).

**Table 2:** Proportion achieving initial and stable RTW within 24 months by job exposure.

Job exposure level (OJI) (*n* = 621,695)	Initial RTW	Stable RTW
Low (OJI 1–3)	147,295 (93.2%)	140,535 (88.9%)
Moderate (OJI 4–7)	212,315 (89.8%)	197,323 (83.4%)
High (OJI 8–10)	201,517 (88.7%)	185,426 (81.6%)
**Total**	561,127 (90.3%)	523,284 (84.2%)

OJI: Overall Job Exposure Index.

In Cox proportional hazards regression models, both moderate and high job exposure were associated with a lower likelihood of initial RTW compared with low exposure. Model 1 was unadjusted, Model 2 adjusted for sociodemographic characteristics, and Model 3 additionally adjusted for work- and health-related characteristics (see Methods). These associations were statistically significant in the unadjusted model (Model 1) and remained largely unchanged after adjustment for sociodemographic characteristics (Model 2). Further adjustment for work- and health-related characteristics (Model 3) resulted in only minor attenuation of the estimates (Table 3).

Further, higher job exposure was significantly associated with a lower likelihood of achieving stable RTW. These associations were present in unadjusted analyses (Model 1) and remained statistically significant after adjustment for sociodemographic variables (Model 2) as well as after additional adjustment for work- and health-related variables (Model 3). Across models, hazard ratios indicated stronger associations for stable RTW than for initial RTW (Table 3).

Supplementary logistic regression analyses examining stable RTW at fixed time points (12 and 24 months after rehabilitation discharge) showed a comparable pattern, with more pronounced differences between low and moderate/high exposure levels compared with the time-to-event analyses (Table S2).

**Table 3:** Hazard ratios for initial and stable return-to-work by job exposure level.

Job exposure level (OJI) (*n* = 621,695)	Model 1 h (95% CI)	Model 2 h (95% CI)	Model 3 h (95% CI)
Initial RTW			
Low (OJI 1–3) *(reference)*			
Moderate (OJI 4–7)	0.874 (0.869–0.880)	0.878 (0.872–0.884)	0.903 (0.897–0.909)
High (OJI 8–10)	0.836 (0.831–0.842)	0.841 (0.835–0.846)	0.872 (0.866–0.878)
**Stable RTW**			
Low (OJI 1–3) *(reference)*			
Moderate (OJI 4–7)	0.850 (0.844–0.856)	0.853 (0.847–0.859)	0.878 (0.872–0.884)
High (OJI 8–10)	0.804 (0.799–0.810)	0.806 (0.800–0.812)	0.837 (0.830–0.843)

OJI: Overall Job Exposure Index, HR: hazard ratio, CI: confidence interval. Model 1: unadjusted. Model 2: adjusted for sociodemographic characteristics. Model 3: additionally adjusted for work- and health- related characteristics. Included variables and their categorisation are shown in Table 1.

**Fig. 1:**
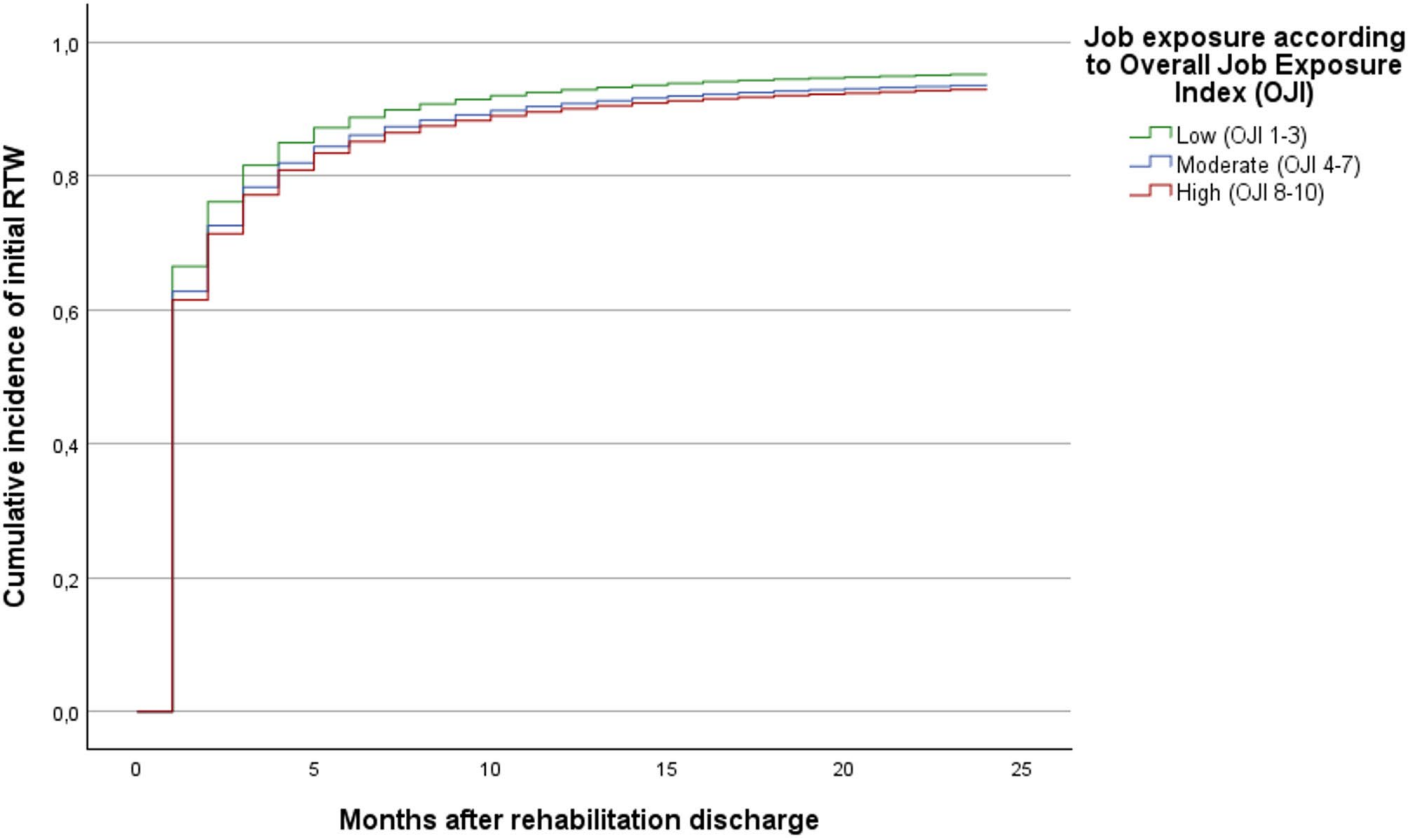
Cumulative incidence of initial RTW by job exposure level. Adjusted for sociodemographic, work- and health-related characteristics (Model 3). Included variables and their categorisation are shown in Table 1.

**Fig. 2:**
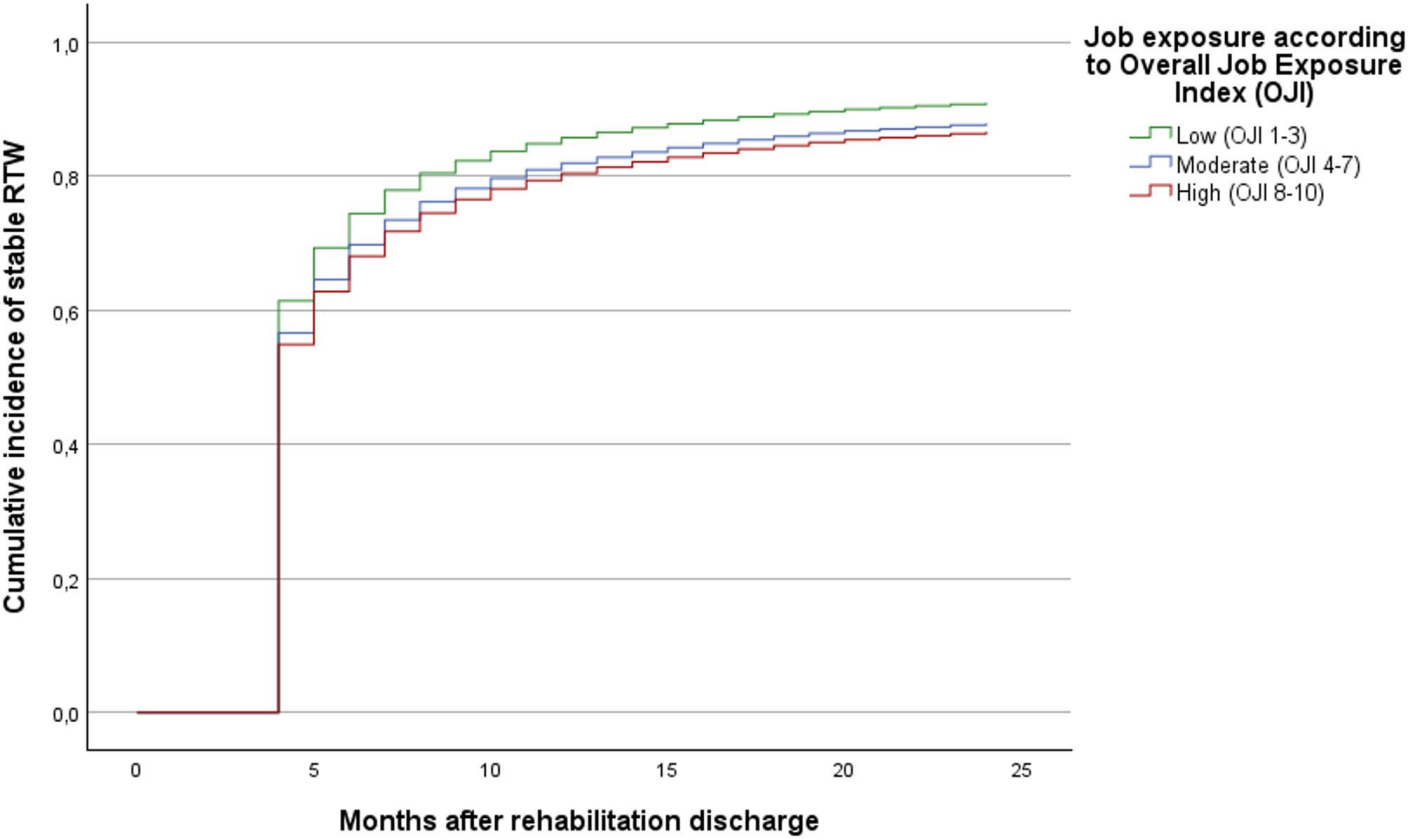
Cumulative incidence of stable RTW by job exposure level. Adjusted for sociodemographic, work- and health-related characteristics (Model 3). Included variables and their categorisation are shown in Table 1.

Cumulative incidence curves (1 − survival functions) illustrate the month-by-month cumulative entry into initial and stable RTW, stratified by job exposure level. Differences between job exposure groups became apparent within the first month after rehabilitation discharge and persisted throughout the 24-month observation period. Although the curves showed some convergence over time, individuals with high job exposure consistently exhibited a lower cumulative probability of RTW compared with those with low exposure. Thus, the cumulative incidence curves complement the Cox regression results by illustrating the early emergence and persistence of exposure-related differences over time (Figs. 1 and 2).

## Discussion

In summary, occupation-based job exposure as measured by the Overall Job Exposure Index (OJI) was consistently associated with both initial and stable RTW within 24 months after medical rehabilitation. Individuals with higher job exposure showed lower RTW rates, delayed entry into employment, and a reduced likelihood of sustained employment. Differences by job exposure level emerged early after rehabilitation discharge and persisted throughout the 24-month follow-up period. Supplementary analyses of stable RTW at 12 and 24 months post-rehabilitation were consistent with the main findings. The largely unchanged estimates across unadjusted and adjusted models indicate that the association between job exposure and return-to-work is robust and not substantially driven by differences in sociodemographic, work-related, or health-related characteristics. To our knowledge, this is the first nationwide study examining occupation-based job exposure in relation to realised initial and sustained return-to-work across the full spectrum of medical rehabilitation indications over a two-year follow-up period.

The RTW rates observed in this study were only slightly higher than those reported in comparable analyses based on German Pension Insurance data. In particular, for orthopaedic rehabilitation – the largest diagnostic group – the difference was approximately 2–3% points^33^. This suggests that the applied inclusion criterion, requiring recent employment prior to rehabilitation, had only a limited impact on the overall level of RTW. However, some restriction of generalisability to populations with weaker labour market attachment or more chronic illness trajectories cannot be fully excluded.

Our findings extend previous research that has primarily focused on return-to-work prognosis at the end of rehabilitation in relation to job exposure. Earlier analyses using the OJI demonstrated that higher job exposure was associated with unfavourable social-medical assessments at discharge, including reduced capacity to work in the last job and increased recommendations for subsequent vocational rehabilitation^27.^ Other studies have largely been restricted to return-to-work outcomes following rehabilitation for specific disease groups, such as injuries or cancer, consistently indicating lower RTW rates among individuals exposed to higher job exposure^20,29^. The present study extends this evidence by adopting a comprehensive approach across the entire rehabilitation system, demonstrating that occupation-based job exposure is not only associated with prognostic assessments at discharge or limited to specific diagnoses, but also with realised employment outcomes over a prolonged follow-up period, independent of diagnosis.

The observed associations align with occupational and social epidemiological research linking physically and psychosocially demanding working conditions to adverse health outcomes, reduced work ability, and premature labour market exit. By leveraging nationwide administrative data, the present analyses provide population-level evidence that these mechanisms remain relevant in the context of medical rehabilitation and subsequent work reintegration. Although the data are embedded in the German social security system, the underlying mechanisms linking demanding working conditions to work participation are likely relevant across comparable high-income labour markets.

Several mechanisms may explain the association between higher job exposure and reduced return-to-work. Individuals in physically or psychosocially demanding occupations may face greater challenges in resuming work tasks after rehabilitation, particularly when residual functional limitations persist. Even when general work ability improves, occupation-specific demands may exceed individual capacity, leading to delayed or unstable reintegration. The early separation of cumulative incidence curves suggests that these effects may occur shortly after discharge, potentially reflecting limited opportunities for workplace accommodation in highly demanding occupations. The persistence of differences over time indicates that such initial disadvantages are not easily compensated. Although the observed hazard ratios were moderate in magnitude, even small relative differences may translate into substantial numbers of affected individuals at the population level given the size of the rehabilitation population.

When interpreting these findings, several strengths and limitations should be considered. A major strength is the use of comprehensive nationwide routine data from the German Pension Insurance, covering a very large cohort of insured persons undergoing medical rehabilitation. This data basis enables robust longitudinal analyses of post-rehabilitation employment trajectories across a wide range of indications and occupational groups^21^. As the German Pension Insurance is the principal provider of medical rehabilitation for persons of working age, the findings are broadly applicable to this population segment, although self-employed individuals and civil servants are underrepresented^19^.

Further strengths include the objective measurement of RTW based on contribution records, reducing reporting bias, and the availability of monthly data allowing differentiation between initial and stable employment. The OJI enabled the integration of occupation-related physical and psychosocial exposures into routine data analyses without additional data collection^34^. Unlike many job exposure matrices restricted to specific occupations or exposure dimensions, the OJI covers the full occupational spectrum and integrates multiple domains of job demands based on a large-scale nationwide survey^25,26^. The index has been validated at national and European levels^34–36^.

Several limitations should also be acknowledged. As an observational study based on routine data, causal conclusions cannot be drawn, and residual confounding by unmeasured factors, such as workplace accommodations, employer characteristics, detailed functional capacity at discharge, or individual health behaviours, cannot be excluded^37^. Although extensive adjustment for sociodemographic, work-related, and health-related characteristics was performed, unmeasured differences in health status or workplace conditions may partly explain the observed associations. In particular, both education and income are closely correlated with occupation, which forms the basis of the OJI. Adjustment for these factors could therefore introduce overadjustment bias, whereas their omission may contribute to residual confounding. In addition, data on educational level was not included as this variable is only incompletely and inconsistently available in the dataset, is not required for administrative purposes, and is not systematically validated. Information on income is limited to individual social insurance-relevant earnings and does not fully capture overall socioeconomic position, including household context.

Job exposure was assessed indirectly using an occupation-based index, which reflects typical rather than individual working conditions^38–40^. In particular, psychosocial job exposures may be captured less precisely than physical demands^35,41^. Misclassification of individual exposure levels is therefore possible and may have attenuated associations. Such non-differential misclassification would likely bias estimates towards the null, suggesting that the true associations may be underestimated rather than overstated. In addition, occupational information reported by employers may contain inaccuracies, particularly for unstable employment histories^22,27^. The aggregation of multiple exposure domains into a single index further limits the ability to identify which specific physical or psychosocial exposures are most relevant for return-to-work outcomes. While some exposure types are likely more influential than others, this cannot be disentangled using the OJI or similar job-exposure-matrix-based approaches, as these reflect average exposure profiles at the occupational level rather than individual working conditions of rehabilitation patients.

Reverse causation appears unlikely, as job exposure was assigned based on occupation prior to rehabilitation; however, long-term selection processes into occupations with different exposure profiles over the life course cannot be fully excluded.

These findings have practical and research implications. Assessing occupation-related job exposures appears relevant for both initial and sustained return-to-work after rehabilitation. In clinical settings, detailed individual assessment of working conditions remains preferable, as it allows rehabilitation professionals to tailor interventions to specific physical and psychosocial demands. Addressing job exposures during rehabilitation may support more sustainable work reintegration.

At the same time, occupation-based indices such as the OJI offer a pragmatic alternative when individual exposure assessment is not feasible, particularly in secondary analyses of administrative data. However, such indices do not provide sufficient detail to guide specific workplace interventions targeting distinct exposure types. Beyond research applications, such indices may inform administrative decision-making and support targeted referral to work-oriented rehabilitation approaches, including work-related medical rehabilitation (MBOR)^42^, especially for individuals in high-exposure occupations.

The findings may also inform preventive strategies aimed at reducing disability pension risk. Established risk models based on routine data do not typically incorporate occupation-specific job exposures^32^. Integrating exposure-based information may enhance risk stratification and support earlier preventive interventions, an issue that warrants further investigation.

In conclusion, in this large nationwide study based on routine administrative data, higher occupation-based job exposure was consistently associated with a lower likelihood of both initial and stable return-to-work after medical rehabilitation. Differences emerged early and persisted over a 24-month follow-up period, underscoring the relevance of work-related demands for long-term rehabilitation outcomes. While individual exposure assessment remains essential at the clinical level, occupation-based indices provide a feasible and informative approach when detailed exposure data are unavailable and may contribute to more targeted rehabilitation and prevention strategies.

## Methods

### Study design and data source

We conducted a retrospective cohort study based on routine administrative data from the German Pension Insurance. The German Pension Insurance is the main provider of medical and vocational rehabilitation for persons of working age in Germany, with the primary aim of maintaining or restoring work ability and preventing long-term disability^19^.

The analyses were based on the Scientific Use File “Completed rehabilitation in the insurance history 2014–2021” provided by the Research Data Centre of the German Pension Insurance^43^. This factually anonymised longitudinal dataset represents a 20% random sample of all medical rehabilitation measures financed by the German Pension Insurance and contains individual-level information on completed medical rehabilitation measures over an eight-year period (2014–2021), including sociodemographic characteristics, occupational information, employment histories, and contribution periods before and after rehabilitation.

## Study population

The study population comprised insured persons aged 18 to 63 years who completed a first medical rehabilitation lasting at least seven days between 2014 and 2019. The year 2019 was chosen as the last inclusion year to ensure a complete follow-up period of 24 months after rehabilitation discharge for all included individuals. Further inclusion criteria were: (1) at least one month of employment subject to compulsory social insurance contributions within the six months prior to rehabilitation application, and (2) valid occupational information documented according to the German Classification of Occupations (*Klassifikation der Berufe*,* KldB*) prior to rehabilitation. In total, 621,695 insured persons met the inclusion criteria and were included in the analyses.

### Occupational information and job exposure assessment

Occupational information in the routine data is coded according to different versions of the KldB. These data are reported by employers as part of the mandatory data transmission process to the social insurance system in accordance with the Data Collection and Transmission Ordinance *(Datenerfassungs- und Übermittlungsverordnung*,* DEÜV)*. Employers are required to submit occupational information at least once per year^44^. For the present analyses, the most recent occupational information available prior to the start of medical rehabilitation was used.

As the Overall Job Exposure Index (OJI) is not available for all historical occupational classifications, occupations originally coded according to KldB 1988 were transformed to KldB 1992 at the level of three-digit occupational orders using established crosswalks^45^. Occupations coded according to KldB 2010 in more recent years were transformed accordingly to ensure compatibility with the job exposure matrices. Previous research has shown that applying the OJI based on KldB 1988 or KldB 2010 yields no substantive differences^22^.

Job exposures were operationalised with the Overall Job Exposure Index (OJI) developed first by Kroll^26^. The OJI is based on 39 items derived from a large-scale nationwide occupational health survey among 20,000 employed persons in Germany^25^ and assigns occupation-specific exposure levels using job-exposure matrices estimated by multilevel modelling adjusted for sex, age, job experience and working hours^34^. A full list of all items included in the index is provided in the Supplementary Material (Table S3). The index captures physical and psychosocial job exposures associated with each occupation and ranges from 1 (lowest exposure) to 10 (highest exposure), representing deciles of the employed population in Germany^26^.

For the present analyses, the OJI was categorised into low (1st–3rd decile), moderate (4th–7th decile), and high (8th–10th decile) job exposure levels. Exposure values were assigned at the most detailed occupational level available; if this was not possible, assignment was performed at the next higher hierarchical level of the KldB^22^.

## Outcome measures

Two return-to-work (RTW) outcomes were defined:

1. Initial RTW was defined as the occurrence of at least one month of employment subject to compulsory social insurance contributions within 24 months after rehabilitation discharge. For descriptive analyses, initial RTW was operationalised as a binary indicator capturing whether an individual achieved at least one such employment month during follow-up.
2. Stable RTW, also referred to as sustained employment, was defined as at least four consecutive months of employment subject to compulsory social insurance contributions within the 24-month follow-up period, in accordance with an established definition of the German Pension Insurance^33^.

Labour market trajectories were reconstructed on a monthly basis using documented compulsory contribution periods, voluntary contribution periods, and creditable periods. Time was modelled in discrete monthly intervals due to the structure of the routine data.

### Statistical analysis

Descriptive analyses were conducted to estimate the proportion of individuals achieving initial RTW and stable RTW within 24 months after rehabilitation discharge, stratified by job exposure level (Table 2). For descriptive comparison with selected previous studies, primarily involving the German Pension Insurance, reporting RTW at predefined follow-up times, supplementary analyses of stable RTW exactly 12 and 24 months after rehabilitation discharge were performed and are presented in the Supplement (Table S1)^33,46^.

Associations between job exposure levels and RTW outcomes were examined using Cox proportional hazards regression models (Table 3). For initial RTW, the event was defined as the first observed RTW event, corresponding to the first month with employment subject to compulsory social insurance contributions during follow-up. For stable RTW, the event was defined as the end of the first episode of four consecutive months of such employment. Time was measured in discrete monthly intervals from rehabilitation discharge until the respective event, reflecting the structure of the routine data.

Hazard ratios (HR) with 95% confidence intervals (95% CI) were estimated for moderate and high job exposure levels compared with low exposure (reference). Three progressively adjusted models were specified:

- Model 1: unadjusted.
- Model 2: adjusted for the following sociodemographic variables: age, sex, citizenship, marital status, and settlement structure of the place of residence according to the classification of districts by the German Federal Institute for Research on Building, Urban Affairs and Spatial Development (BBSR)^47,48^.
- Model 3: additionally adjusted for the following work- and health-related variables: incapacity for work at rehabilitation discharge (physician-assessed), sick leave duration in the 12 months prior to rehabilitation, and disease group at the time of rehabilitation approval.

In addition, to complement the time-to-event analyses and to facilitate comparability with studies focusing on RTW status at predefined follow-up times, separate logistic regression analyses were conducted for stable RTW at 12 months and at 24 months after rehabilitation discharge (Supplement Table S2). In these analyses, stable RTW at the respective time point was treated as a binary outcome. For each follow-up time, three logistic regression models were specified, applying the same stepwise adjustment strategy as in the Cox regression analyses.

The categorisation of the variables included in the models, as shown in Table 1, is based on the structure of the Scientific Use File and follows an established approach^33^.

To illustrate the temporal dynamics of RTW beyond the summary estimates provided by the Cox and logistic regression models, cumulative incidence curves (1 − survival) were used to depict the month-by-month cumulative entry into initial and stable RTW over the 24-month follow-up period by job exposure level.

For the statistical analyses, SPSS (IBM Corp., Armonk, NY, USA) and R (R Foundation for Statistical Computing, Vienna, Austria) were used.

### Ethical considerations and reporting standards

This study was performed in line with the principles of the Declaration of Helsinki. Approval was granted by the Ethics Committee of the Charité – Universitätsmedizin Berlin (EA1/207/18). The study is based on anonymised routine administrative data (Scientific Use File) provided by the Research Centre of the German Pension Insurance under a formal data use agreement. As the data were fully anonymised and no contact with individual insured persons occurred, individual informed consent was not required. The study was conducted in accordance with the principles of Good Epidemiological Practice and the Good Practice of Secondary Data Analysis^49,50^. Reporting follows the German Reporting Standard for Secondary Data Analyses (STROSA)^51^.

## Supplementary Information

Below is the link to the electronic supplementary material.

Supplementary Material 1

## Data Availability

The Scientific Use File analysed in this study is available from the Research Data Centre of the German Pension Insurance upon reasonable request (https://fdz-rv.de/en).
