# Supplementary material for: Higher job exposures are associated with reduced return-to-work two years after rehabilitation in a nationwide cohort study based on German Pension Insurance data
Source: Sci Rep. 2026 Jun 2;16:16985. doi: 10.1038/s41598-026-55323-0 (PMC13230846; doi:10.1038/s41598-026-55323-0)
Supplement: Supplementary file 1 — Supplementary Material 1 [file 41598_2026_55323_MOESM1_ESM.docx]

**Higher job exposures are associated with reduced return to work two years after rehabilitation in a nationwide cohort study based on German Pension Insurance data**

**Supplement**

**Table S1.** Proportion achieving stable return to work 12 and 24 months post-rehabilitation by job exposure level

| **Job exposure level (OJI)**  (n=621,695) | **Stable RTW  12 months  post-rehabilitation** | **Stable RTW  24 months  post-rehabilitation** |
| --- | --- | --- |
| Low (OJI 1-3) | 123,412 (78.1 %) | 117,612 (74.4 %) |
| Moderate (OJI 4-7) | 167,969 (71.0 %) | 161,723 (68.4 %) |
| High (OJI 8-10) | 155,651 (68.5 %) | 150,292 (66.2 %) |
| **Total** | 447,032 (71.9 %) | 429,627 (69.1 %) |

OJI: Overall Job Exposure Index

**Table S2.** Odds ratios for stable return to work 12 and 24 months post-rehabilitation by job exposure level

| **Job exposure level (OJI)** (n=621,695) | **Model 1 OR (95% CI)** | **Model 2 OR (95% CI)** | **Model 3 OR (95% CI)** |
| --- | --- | --- | --- |
| **Stable RTW at 12 months** |  |  |  |
| Low (OJI 1-3) *(reference)* |  |  |  |
| Moderate (OJI 4-7) | 0.688 (0.678–0.699) | 0.690 (0.680–0.701) | 0.732 (0.720–0.744) |
| High (OJI 8-10) | 0.611 (0.602–0.620) | 0.605 (0.596–0.614) | 0.655 (0.644–0.666) |
| **Stable RTW at 24 months** |  |  |  |
| Low (OJI 1-3) *(reference)* |  |  |  |
| Moderate (OJI 4-7) | 0.744 (0.733–0.755) | 0.737 (0.726–0.748) | 0.780 (0.768–0.793) |
| High (OJI 8-10) | 0.673 (0.663–0.682) | 0.649 (0.639–0.659) | 0.700 (0.689–0.711) |

OJI: Overall Job Exposure Index; OR: odds ratio; CI: confidence interval; RTW: return to work. Model 1: unadjusted. Model 2: adjusted for sociodemographic characteristics. Model 3: additionally adjusted for work- and health- related characteristics. Included variables and their categorisation are shown in Table 1.

**Table S3.** Overall Job Exposure Index: List of included items from the BIBB/BAuA Employment Survey

|  | exposure domain | item |
| --- | --- | --- |
| physical job exposures | ergonomic exposures | work standing up |
|  |  | work at least one hour continuously in a seated position |
|  |  | lifting and carrying of loads of more than 20 kg (women: 10 kg) |
|  |  | work in a stooped, squatting or kneeling position, or overhead |
|  | environmental exposures | work with smoke, dust, or gases and vapours |
|  |  | work under cold, heat, moisture, humidity or draughts |
|  |  | working with oil, grease, or dirt |
|  |  | work with strong vibrations, shocks and oscillations felt in the body |
|  |  | work in bright light or with poor or insufficient illumination |
|  |  | handling dangerous substances |
|  |  | wearing protective clothing or equipment |
|  |  | working under noise |
|  |  | work in a place where people smoke |
| psychosocial job exposures | psychological exposures | working under strong deadline or performance pressure |
|  |  | being confronted with new tasks at work |
|  |  | improving existing procedures or trying something new |
|  |  | being disturbed or interrupted at work |
|  |  | prescribed output targets or time limits |
|  |  | being required to perform tasks not learned or not mastered |
|  |  | monitoring different types of work or processes at the same time |
|  |  | even a small mistake or inattention may result in major financial losses |
|  |  | working at the limits of one’s capabilities |
|  |  | working very fast |
|  | social exposures | planning and scheduling of own work |
|  |  | influence on the amount of work assigned |
|  |  | deciding when to take a break |
|  |  | feeling that the job is important |
|  |  | not being informed in time about major decisions, changes or future plans |
|  |  | not receiving all necessary information to carry out tasks properly |
|  |  | feeling part of a workplace community |
|  |  | perceived cooperation with colleagues |
|  |  | receiving help and support from colleagues when needed |
|  |  | receiving help and support from the immediate supervisor when needed |
|  | time-related exposures | working regularly ≥48 hours per week |
|  |  | standby duty or on-call duty |
|  |  | work on Saturdays |
|  |  | work on Sundays and holidays |
|  |  | night shifts |
|  |  | shift work |

Categorisation and presentation of items based on Ref. 26 and 27. English translation of the original German version adapted from Ref. 25**.**
